# Multimodal imaging of migrating gallbladder mucocele in a Chihuahua

**DOI:** 10.1111/jsap.13886

**Published:** 2025-06-27

**Authors:** F. Valeri, N. Nisini, G. Angeli, E. Lepri, M. Brescia, D. Caivano

**Affiliations:** ^1^ Department of Veterinary Medicine University of Perugia Perugia Italy

**FIG 1:**
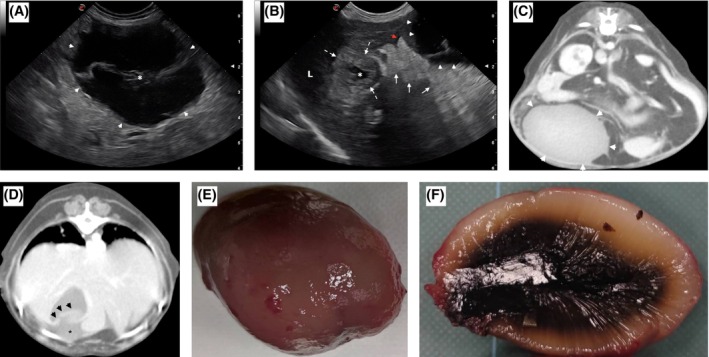
(A) Right sagittal, transabdominal ultrasonographic image of the migrating gallbladder mucocele (white arrowheads) diffusely hypoechoic with an irregular‐shaped hyperechoic centre (asterisk). (B) Right sagittal, transabdominal ultrasonographic image of the liver and gallbladder. Collapsed gallbladder lumen (asterisk), thickened gallbladder wall (dashed line), surrounding fat markedly hyperechoic (white arrows) and a very small amount of free fluid (red arrowhead) are evident. The mucocele can be partially seen (white arrowheads). L = liver. (C) Transverse computed tomographic scan of the gallbladder mucocele at the level of L1 (white arrowheads). (D) Transverse computed tomographic scan of the gallbladder at the level of T10. The gallbladder wall was thickened and irregular (black arrowheads). A tear in the gallbladder wall with content extravasation was evident (asterisk). (E) The migrating gallbladder mucocele after being removed surgically and (F) its section.

A 12‐year‐old 3.7‐kg intact female Chihuahua was evaluated because of a 2‐day history of abdominal pain and inappetence. The dog showed depression and abdominal pain. Haematological and serum biochemical analyses revealed neutrophilic leucocytosis, high activities of aspartate aminotransferase and alkaline phosphatase levels. Abdominal ultrasonography (US) showed a well‐defined, oval‐shaped mass (4.7 cm × 4 cm), caudal to the liver (Fig 1A). The mass was diffusely hypoechoic with an irregular‐shaped hyperechoic centre, and no blood flow was detected by colour Doppler study. The gallbladder wall was thickened, with a collapsed lumen and hyperechoic surrounding fat (Fig 1B). A small amount of free abdominal fluid was detected. Computed tomography (CT) confirmed the presence of an oval‐shaped, non‐contrast‐enhancing mass (6.1 cm × 4.1 cm) in the cranial abdomen caudally to the liver (Fig 1C). The mass appeared with a homogeneous density except for a slightly hyperdense nucleus. The gallbladder wall was thickened and irregular with inward deviation, with external hypodense material visible (Fig 1D). A presumptive diagnosis of migrating gallbladder mucocele secondary to gallbladder rupture was made. At surgery, an ovoidal mass with a smooth surface, firm‐elastic consistency and blackish‐brown colour, adjacent to the liver, was found and removed. Surgical resection of the gallbladder was also performed. The dog recovered uneventfully. Histologically, the gallbladder epithelium was hyperplastic with papillary intraluminal projection, intramural cysts filled with bluish mucoid substance and diffuse lymphoplasmacytic infiltration in the lamina propria. Focally, the gallbladder wall was haemorrhagic and necrotic with calcification. The intra‐abdominal mass was composed of a similar bluish mucoid material. Migrating gallbladder mucocele is a rare condition and cannot be excluded based on clinical and laboratory findings alone. Multimodal imaging (US/CT) provided a more accurate diagnosis, allowing for the documentation of the characteristic pattern of the mucocele and evidence of gallbladder rupture.

